# Serum and Urinary Soluble α-Klotho as Markers of Kidney and Vascular Impairment

**DOI:** 10.3390/nu15061470

**Published:** 2023-03-18

**Authors:** Julia Martín-Vírgala, Sara Fernández-Villabrille, Beatriz Martín-Carro, Isaac Tamargo-Gómez, Juan F. Navarro-González, Carmen Mora-Fernández, Laura Calleros, Elena Astudillo-Cortés, Noelia Avello-Llano, Guillermo Mariño, Adriana S. Dusso, Cristina Alonso-Montes, Sara Panizo, Jorge B. Cannata-Andía, Manuel Naves-Díaz, Natalia Carrillo-López

**Affiliations:** 1 Bone and Mineral Research Unit, Instituto de Investigación Sanitaria del Principado de Asturias (ISPA), Hospital Universitario Central de Asturias (HUCA), Universidad de Oviedo, 33011 Oviedo, Spain; 2 Redes de Investigación Cooperativa Orientadas a Resultados en Salud (RICORS), RICORS2040 (Kidney Disease), 28040 Madrid, Spain; 3 Departamento de Biología Funcional, Facultad de Medicina, Universidad de Oviedo, 33006 Oviedo, Spain; 4 Instituto Universitario de Oncología (IUOPA), 33006 Oviedo, Spain; 5 Autophagy and Metabolism Lab, Instituto de Investigación Sanitaria del Principado de Asturias (ISPA), 33011 Oviedo, Spain; 6 Unidad de Investigación, Hospital Universitario Nuestra Señora de Candelaria, 38010 Santa Cruz de Tenerife, Spain; 7 Servicio de Nefrología, Hospital Universitario Nuestra Señora de Candelaria, Instituto de Tecnologías Biomédicas, Universidad de La Laguna, 38200 San Cristóbal de La Laguna, Spain; 8 Department of Systems Biology, Physiology Unit, Universidad de Alcalá, 28801 Madrid, Spain; 9 Instituto Ramón y Cajal de Investigación Sanitaria (IRYCIS), 28034 Madrid, Spain; 10 Fundación Renal Iñigo Álvarez de Toledo (FRIAT), 28003 Madrid, Spain; 11 Division of Nephrology, Hospital Universitario Central de Asturias (HUCA), 33011 Oviedo, Spain; 12 Laboratorio de Medicina, Hospital Universitario Central de Asturias (HUCA), 33011 Oviedo, Spain; 13 Division of Endocrinology, Metabolism and Lipid Research, Washington University School of Medicine, St. Louis, MO 63110, USA; 14 Departamento de Medicina, Universidad de Oviedo, 33006 Oviedo, Spain

**Keywords:** sKlotho, chronic kidney disease, osteogenic differentiation, serum CKD-MBD biomarkers, vascular calcification

## Abstract

This study was designed to investigate the controversy on the potential role of sKlotho as an early biomarker in Chronic Kidney Disease–Mineral Bone Disorder (CKD-MBD), to assess whether sKlotho is a reliable marker of kidney α-Klotho, to deepen the effects of sKlotho on vascular smooth muscle cells (VSMCs) osteogenic differentiation and to evaluate the role of autophagy in this process. Experimental studies were conducted in CKD mice fed a normal phosphorus (CKD+NP) or high phosphorus (CKD+HP) diet for 14 weeks. The patients’ study was performed in CKD stages 2–5 and in vitro studies which used VSMCs exposed to non-calcifying medium or calcifying medium with or without sKlotho. The CKD experimental model showed that the CKD+HP group reached the highest serum PTH, P and FGF23 levels, but the lowest serum and urinary sKlotho levels. In addition, a positive correlation between serum sKlotho and kidney α-Klotho was found. CKD mice showed aortic osteogenic differentiation, together with increased autophagy. The human CKD study showed that the decline in serum sKlotho is previous to the rise in FGF23. In addition, both serum sKlotho and FGF23 levels correlated with kidney function. Finally, in VSMCs, the addition of sKlotho prevented osteogenic differentiation and induced autophagy. It can be concluded that serum sKlotho was the earliest CKD-MBD biomarker, a reliable indicator of kidney α-Klotho and that might protect against osteogenic differentiation by increasing autophagy. Nevertheless, further studies are needed to investigate the mechanisms of this possible protective effect.

## 1. Introduction

Chronic Kidney Disease (CKD) is a state of decreased kidney function characterized by vascular and bone disturbances, known as Chronic Kidney Disease–Mineral Bone Disorder (CKD-MBD) [1]. The progressive reduction in renal function leads to an increase in fibroblast growth factor 23 (FGF23) and a decrease in phosphate (P) excretion, calcitriol synthesis and also soluble α-Klotho (sKlotho). These changes underlie the pathogenesis of CKD-MBD, negatively impacting the bone and cardiovascular systems [2,3].

The disturbances in calcitriol, P and calcium (Ca) homeostasis are late events in the course of CKD-MBD; on the contrary, the increase in FGF23 and the changes in sKlotho occur in earlier stages of CKD [4,5]. FGF23 can be accurately measured in serum reflecting what happens in bone, where most of the FGF23 is produced. In the kidney, α-Klotho acts as a coreceptor for FGF23 receptor, so FGF23 is able to induce phosphaturic actions [6]. Kidney α-Klotho can be cleaved off by proteases [7] and released into the bloodstream and urine, where it can be measured [8,9]. Nevertheless, there is great controversy on the value of serum and urinary sKlotho as indicators of the α-Klotho content in the kidney, where it is mainly expressed [10,11]. Previous studies do not reach any solid conclusion on sKlotho being used as a CKD biomarker and there is lack of standardized techniques to assess sKlotho [9,12]. Furthermore, it is difficult to explain the presence of sKlotho in the urine, which makes it more complex to explain its values [13,14].

Vascular impairment in CKD is often diagnosed when the damage is already stablished and no early biomarkers are known [15,16]. The reduction in sKlotho has been associated to progression of CKD [17] and to increase in vascular calcification (VC) [8,17], though these statements have been controversial. Besides, the systemic effects of sKlotho are still poorly understood [18,19], although it has been suggested that autophagy might be a mechanism of action of sKlotho [20]. In fact, autophagy might be a defensive mechanism against vascular damage in CKD [21,22].

This study was designed to (a) confirm the potential role of sKlotho as an early biomarker compared with other CKD-MBD biomarkers, (b) to assess whether sKlotho is a reliable indicator of kidney α-Klotho content, (c) to deepen the effects of sKlotho on vascular smooth muscle cells (VSMCs) osteogenic differentiation and (d) to evaluate if autophagy could be involved in the actions of sKlotho.

## 2. Materials and Methods

In the study, three different models were used: (A) mice with mild CKD, (B) patients with CKD stages 2–5 and, (C) vascular smooth muscle cells (VSMCs).

### 2.1. Mice Model with Mild CKD

#### 2.1.1. Experimental Design

To induce CKD, two and a half-month-old male FVB/N mice (*n* = 13) underwent 75% kidney mass reduction (nephrectomy, NX) using two-step surgical intervention. The first intervention consisted of the decapsulation of the kidney and afterwards a bipolar resection of the left kidney by cauterization. One week later, the right kidney was totally removed. The anesthetic method used was isoflurane by inhalation. One week after the second intervention, they were divided into two experimental groups: One group received a normal phosphorus (NP) diet (0.6% P and 0.6% Ca, Panlab, Spain; *n* = 6) and the other group received a high phosphorus (HP) diet (0.9% P, 0.6% Ca, *n* = 7). A third group of Sham-operated mice fed an NP diet (*n* = 8) was used as reference group. All mice received the described food for 14 weeks, thereafter they were placed in metabolic cages for 24-h urine collection prior to sacrifice. At sacrifice, blood and urine samples, aortas and kidneys were collected and stored at −80 °C until analysis.

All protocols were approved by the Ethics Committee for laboratory animals of the Oviedo University (PROAE 03/2017).

#### 2.1.2. Biochemical Markers

Blood chemistries’ analysis utilized Quanti-Chrom™ Specific Assay Kits (Bioassay Systems) for serum creatinine, BUN, Ca and P. Specific ELISA kits quantified the serum intact parathyroid hormone (PTH; Immutopics, Inc., San Clemente, CA, USA), intact FGF23 (Immutopics, San Clemente, CA, USA), calcitriol (1,25(OH)2D3; Elabscience Biotechnology Inc., Houston, TX, USA) and sKlotho (Cusabio Technology LLC, Houston, TX, USA).

In urine samples, Quanti-Chrom™ Specific Assay Kits (Bioassay Systems, Hayward, CA, USA) were used to measure Ca and P, and ELISA assays were performed to quantify albumin (Bethyl Laboratories, Inc., Montgomery, MD, USA) and sKlotho levels (IBL Co., Männedorf, Switzerland).

#### 2.1.3. Kidney Interstitial Fibrosis and Immunohistochemistry for Kidney α-Klotho and Wnt/β-Catenin Signaling

Kidney interstitial fibrosis was determined using Masson’s Trichrome staining (standard protocol) in deparaffined 2.5 µm sections of the kidney. It was quantified by using an optical microscope (model DMRXA2; Leica Microsystems, Wetzlar, Germany) coupled to a digital video camera (model Dc-100; Leica Microsystems) and to an image analysis system with specific software (ImageJ 1.53k). Measurements were performed by a technician blinded to experimental groups and interventions. Results were expressed as the percentage of blue area of the total kidney area excluding peri-glomerular and peri-vascular blue staining.

The expression analysis by immunohistochemistry of kidney α-Klotho and non-phospho (active) β-catenin were performed using the EnVision FLEX Mini Kit (K8024, DAKO, Glostrup, Denmark) and Dako Autostainer systems. Paraffin-embedded kidneys (2.5 µm) were deparaffinized, rehydrated and underwent epitope retrieval (HIER) by heat induction at 95 °C for 20 min and pH 9 (GV804DAKO, DAKO, Glostrup, Denmark) in the Pre-Treatment Module, PT-LINK (DAKO, DAKO, Glostrup, Denmark). Kidney sections were incubated with Klotho and non-phospho (active) β-catenin antibody (Table S1 (Supplementary Materials)) diluted in EnVision™ FLEX Antibody Diluent (K8006, DAKO, Glostrup, Denmark) for 60 min, after blocking endogenous peroxidase with EnVision™ FLEX Peroxidase-Blocking Reagent (DM821, DAKO, Glostrup, Denmark). The signal was detected using diaminobenzidine chromogen as substrate (DM823, DAKO, Glostrup, Denmark) in EnVision™ FLEX Substrate Buffer (DM823, DAKO, Glostrup, Denmark) and Dako EnVision™ FLEX/HRP (DM822, DAKO, Glostrup, Denmark). Sections were counterstained with hematoxylin. Appropriate negative controls were also tested. After the whole process, sections were dehydrated and mounted with permanent medium (Dako mounting medium, CS703, DAKO, Glostrup, Denmark).

#### 2.1.4. Soft Tissue Quantification of Ca Deposition

Two methods were used to assess soft tissue Ca deposition: Von Kossa [23] and Alizarin red [24] staining. Von Kossa staining: Kidney sections of 2.5 µm were deparaffinized, hydrated and stained following the Von Kossa method [23]. Alizarin red staining: 2.5 µm aortic paraffin sections were deparaffinized and rehydrated and Ca deposits were stained with Alizarin red [24]. The quantification of Ca deposition followed the same protocol described for the quantification of kidney fibrosis. Results were expressed as the percentage of calcified area compared to the total area.

#### 2.1.5. Aortic Immunofluorescence for Autophagy Analysis

Aortic paraffin sections of 2.5 µm were incubated overnight with anti-LC3BII antibody (Table S1) at 4 °C on a humidifying chamber. LC3BII area was measured by fluorescent positive cells (%), using ImageJ.

### 2.2. Patients CKD Stages 2–5 and Controls

A total of 43 CKD patients (25 men and 18 women) and 38 sex- and age-matched controls (18 men and 20 women) were studied. The average age was 66.65 ± 8.58 in CKD patients and 66.50 ± 4.63 in controls. The CKD patients were classified according to their estimated glomerular filtration rate (eGFR) into four groups: CKD-2/3a, CKD-3b, CKD-4 and CKD-5 [25]. The exclusion criteria were (a) diabetes mellitus, (b) abdominal aneurism or intermittent claudication, (c) previous carotid surgery, (d) concomitant immune-mediated disease or cancer diagnosis, (e) ongoing immunosuppressive treatment, (f) recent or current infection or (g) pregnancy. All protocols were approved by the Ethics Committee for drug research of the Principado de Asturias (CEImPA 140/19).

Age, sex, body mass index (BMI), blood and urinary parameters were collected. Creatinine was determined by isotope dilution mass spectrometry (IDMS; Roche Diagnostics, Basel, Switzerland). Total proteins, Ca and P were determined by photometry (Roche Diagnostics, Basel, Switzerland), PTH using an electrochemiluminescence immunoassay (ECLIA; Roche Diagnostics, Basel, Switzerland) and FGF23 and calcitriol were analyzed with a chemiluminescence immunoassay (CLIA; DiaSorin, Saluggia, Italy). Finally, the concentration of sKlotho, both in serum and urine, was evaluated by a specific ELISA assay (Immuno-Biological Laboratories, IBL, Männedorf, Switzerland).

### 2.3. In Vitro Studies

#### 2.3.1. Experimental Design

Two different in vitro approaches were used (i) to analyze the possible effect of sKlotho on VSMCs muscular and osteogenic phenotype changes towards calcification after 24 and 72 h of incubation in calcifying (CM) and non-calcifying medium (non-CM) and (ii) to study if autophagy plays a role in the effect of sKlotho on the above mentioned VSMCs’ phenotypic changes. Primary VSMC cultures were obtained from aortas of normal non-manipulated 2-month-old Wistar rats using explants as previously described [26]; cells between passages 2 and 8 were used.

#### 2.3.2. Effect of sKlotho on VSMC Phenotypic Changes towards Osteogenic Differentiation

VSMCs were seeded at 100,000 cell/cm^2^ in 6-well plates (Corning Costar, Glendale, CA, USA) and grown in Dulbecco Modified Eagle Medium (DMEM) supplemented with 10% fetal bovine serum (FBS) and 1% penicillin/streptomycin (Lonza, Alsace, France) to sub-confluence. Cells were exposed to Non-CM (1.8 mM Ca, 1 mM), CM (1.8 mM Ca, 3 mM P) [27,28,29] or CM + sKlotho (50 ng/mL, R&D Systems, Minneapolis, MN, USA, [30]). This recombinant protein has proven to be biologically active [30,31] and effective in osteoblast cell cultures [30]. The experiments were performed in triplicates for each condition, cells were exposed to CM and Non-CM during 24 or 72 h, replacing the culture media every day. Cells were collected for Ca content quantification and total RNA extraction and stored at −80 °C until analysis.

#### 2.3.3. sKlotho and VSMCs Phenotypic Signaling towards Vascular Calcification and Autophagy

To characterize if sKlotho induces autophagy, primary VSMCs and A7r5 (ATCC) cells were seeded in 96-well imaging plates (1,000 cells/well; BD Falcon, Sparks, NV, USA) for immunofluorescence and in 6-well plates to obtain protein for Western blot.

The primary VSMCs and A7r5 cells were exposed during 24 h to Non-CM, CM and CM+sKlotho. To induce autophagy, after 20 h (4 h before the end of the experiment), cells were exposed to nutrient free media (Nf). Immunofluorescence (LC3B-II puncta) and Western blot [32] (LC3B-II/LC3B-I ratio) were performed and quantified.

#### 2.3.4. Quantification of Ca Deposition in the VSMCs

VSMCs were homogenized in 0.6 N HCl and gently shaken at 4 °C for 24 h. After sample centrifugation, Ca was quantified in the supernatant by the o- cresolphthalein complexone method [27] (Sigma, St. Louis, MO, USA). The pellet was resuspended in lysis buffer (125 mM Tris, 2% SDS, pH 6.8) for protein extraction and quantification by the Lowry method (Bio Rad, Hercules, CA, USA). Ca content was normalized to total cell protein and expressed as μg Ca/mg protein.

#### 2.3.5. Evaluation of Autophagy Using LC3B-II Puncta VSMCs Immunofluorescence

Primary VSMCs and A7r5 were fixed with 4% paraformaldehyde. Wells were blocked in 5% bovine serum albumin (BSA) in Tris-buffered saline with Tween 20 buffer (TBST) for 30 min at room temperature and incubated with anti-LC3B (Table S1) diluted in 3% BSA in TBST overnight at 4 °C. After three washes in PBS, cells were incubated for 40 min at room temperature with secondary antibodies, thoroughly washed in PBS three times and stained with DAPI for nuclear staining.

### 2.4. Common Analytical and Technical Procedures Used in the Studies

#### 2.4.1. RNA Extraction, cDNA Synthesis and Quantitative PCR

Total RNA from kidney, aorta and primary VSMCs was extracted using TRI reagent (Sigma-Aldrich) and 2µg of total RNA was retro-transcribed using the High Capacity cDNA Reverse Transcription Kit (Applied Biosystems, Waltham, MA, USA). Quantitative real time PCR (qPCR) was performed using pre-developed assays (Table S2) in the Stratagene Mx3005P QPCR System (Agilent Technologies, Santa Clara, CA, USA). All reactions were performed in triplicate. Relative gene expression was quantified by the ΔΔCT method [33].

#### 2.4.2. Western Blot Analysis

Total proteins from kidney and primary VSMCs were extracted using a standard RIPA buffer with protease inhibitors and 0.2 mM sodium orthovanadate (Sigma-Aldrich, St. Louis, MO, USA).

For kidney samples, a total of 40 μg of protein was electrophoresed on 8% SDS-PAGE minigels and transferred to a Hybond™ P membrane (GE Healthcare, Chicago, IL, USA). Primary antibodies are specified in Table S1. Chromogenic detection was performed with ECL Western Blotting Detection Kit (Amersham Biosciences, Amersham, UK) and ChemiDoc Gel Imaging system (Bio-Rad, Hercules, CA, USA) and quantified using Quantity One1-D Analysis Software (Bio-Rad, Hercules, CA, USA).

For primary VSMC, a total of 15 μg of protein sample was electrophoresed on 15% SDS polyacrylamide gels and electro-transferred onto polyvinylidene difluoride (PVDF) membranes (Millipore, Burlington, VT, USA). Primary antibodies are specified in Table S1. Membranes were incubated with the corresponding secondary antibody and were developed with Immobilon Western Chemiluminescent HRP substrate (Millipore, Burlington, VT, USA) by using Odyssey. Fc Imaging System (LI-COR, Lincoln, NE, USA). Primary antibodies are specified in Table S1.

### 2.5. Statistical Analysis

Results are expressed as median (interquartile range) or mean ± standard deviation as indicated. Statistical comparisons between groups were performed using the Kruskal–Wallis test (non-parametric analysis) or ANOVA (parametric analysis) and Dunn or Tukey tests (to calculate specific differences between every group) as post hoc analysis. A *p* value lower than 0.05 was considered statistically significant. All calculations used the statistical analysis package R (3.6.1 version).

## 3. Results

### 3.1. Mice Model with Mild CKD

#### 3.1.1. Biochemical Parameters and Kidney Function

After 14 weeks, both groups of nephrectomized mice (CKD+NP and CKD+HP) showed significantly higher serum creatinine and BUN levels compared to the reference group (Sham-operated mice+NP), without differences between the CKD groups. In addition, the CKD+HP mice showed lower body weight, higher serum levels of P, PTH and FGF23 and lower levels of serum sKlotho (Table 1). A negative correlation between serum sKlotho and creatinine, BUN, Ca, PTH, FGF23 and phosphaturia were found (Creatinine: r = −0.690, *p* = 0.0005; BUN: r = −0.573, *p* = 0.007; Ca: r = −0.453, *p* = 0.039; PTH: r = −0.549, *p* = 0.012; FGF23: r = −0.471, *p* = 0.031 and phosphaturia: r = −0.468, *p* = 0.037).

In the urine, the CKD+HP group showed lower levels of Ca and sKlotho and higher levels of P and urinary albumin compared to the CKD+NP group (Table 1). There was no significant correlation between urinary sKlotho, and the serum and urinary parameters analyzed in the study.

#### 3.1.2. Kidney Fibrosis, α-Klotho Expression and Wnt/β-Catenin Signaling

**Figure 1:**
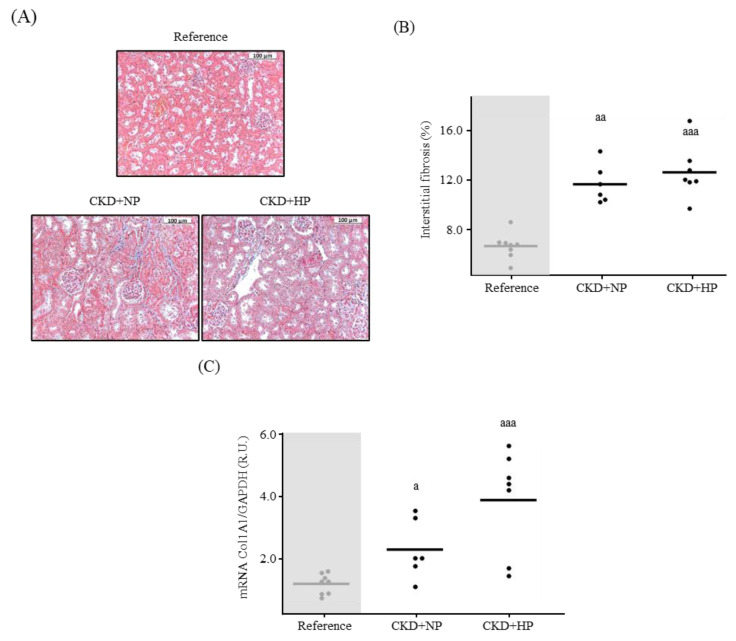
In vivo effects of chronic kidney disease (CKD) and dietary phosphorus intake on kidney fibrosis. (**A**) Representative images for kidney interstitial fibrosis and (**B**) quantification from Sham-operated (Reference) and CKD mice fed a normal phosphorus (NP) or high phosphorus (HP) diet for 14 weeks. Each inset indicates the relative scale in µm. Mean and individual mice values are shown. ^aa^ *p* < 0.01 and ^aaa^ *p* < 0.005 vs. Reference. (**C**) Kidney Collagen 1 mRNA levels. All values are expressed relative to Reference group. Mean and individual mice values are shown. ^a^ *p* < 0.05 and ^aaa^ *p* < 0.005 vs. Reference. R.U., relative units vs. Sham+NP. GAPDH was used as housekeeping gene.

The Masson’s Trichrome staining showed significantly greater interstitial kidney fibrosis in the CKD groups compared to the reference group (Reference group: 6.75 ± 1.18% vs. CKD groups: 12.98 ± 2.14%, *p* = 0.0007; Figure 1A,B). A significant negative correlation between kidney fibrosis and serum sKlotho was found in the three experimental groups (r = −0.756; *p* = 0.00007). No correlation was found when the reference group was excluded (r = −0.180; *p* = 0.556). The kidney collagen 1 gene expression followed a similar pattern achieving the highest mRNA levels in the CKD+HP group (Figure 1C).

**Figure 2:**
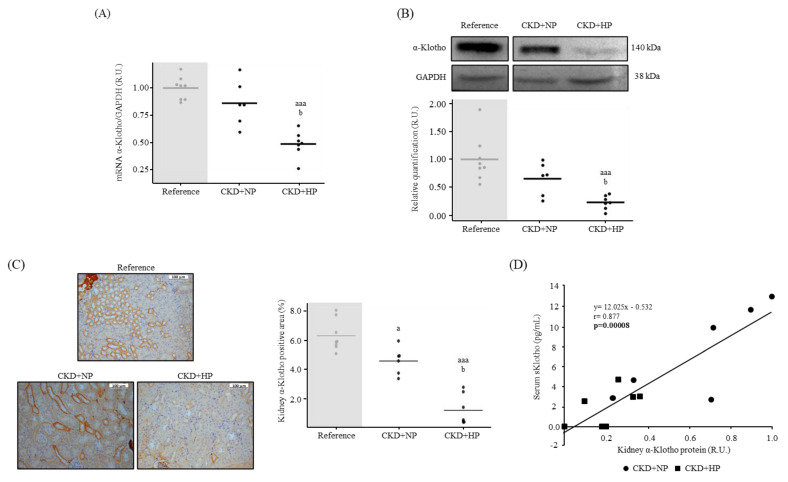
In vivo effects of chronic kidney disease (CKD) and dietary phosphorus intake on kidney α-Klotho expression and its correlation with sKlotho (**A**) Kidney α-Klotho mRNA levels from Sham-operated fed normal phosphorus (NP) (Reference) and CKD mice fed NP or high phosphorus (HP) diet for 14 weeks. All values are expressed relative to reference group. Mean and individual mice values are shown. ^aaa^ *p* < 0.005 vs. Reference, ^b^ *p* < 0.05 vs. CKD+NP. R.U., relative units vs. Reference. (**B**) Representative image of Western blot analysis and relative quantification. All values are expressed relative to the reference group. Horizontal lines depict mean values. ^aaa^ *p* < 0.005 vs. Reference, ^b^ *p* < 0.05 vs. CKD+NP. R.U.: Relative Units vs. reference group. (**C**) Representative images of kidney α-Klotho and its quantification. Each inset indicates the relative scale in µm. ^a^ *p* < 0.05 vs. Reference, ^aaa^ *p* < 0.005 vs. Reference, ^b^ *p* < 0.05 vs. CKD+NP. (**D**) Correlation between kidney α-Klotho protein expression and soluble Klotho (sKlotho) levels within CKD groups. Each shape represents, CKD+NP (●) and CKD+HP (■) values. GAPDH was used as housekeeping gene or loading control.

Kidney mRNA α-Klotho expression was significantly lower in the CKD+HP group compared to the reference and the CKD+NP groups (Figure 2A). Klotho protein expression (Western blot and immunohistochemistry), followed the same pattern as gene expression (Figure 2B,C), showing significantly lower levels in the CKD+HP mice compared to the reference and the CKD+NP mice. Serum sKlotho strongly and positively correlated with kidney α-Klotho in both CKD groups (r = 0.880; *p* = 0.00008, Figure 2D). The correlation was also observed when the reference group was added (r = 0.600; *p* = 0.004). In the CKD groups, urinary sKlotho decreased (Table 1), but it did not show any correlation with kidney α-Klotho (r = 0.194, *p* = 0.40).

**Figure 3:**
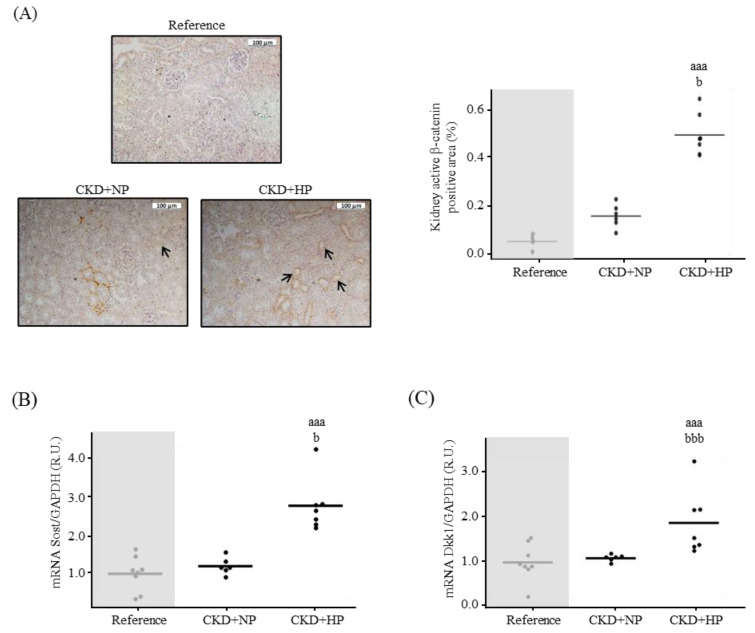
In vivo effects of chronic kidney disease (CKD) and dietary phosphorus intake on components of the Wnt/β-catenin pathway in the kidney. (**A**) Representative images of kidney active β-catenin and its quantification from Sham (Reference) and CKD mice fed normal (NP) or high phosphorus (HP) diet for 14 weeks. Each inset indicates the relative scale in µm. Kidney (**B**) Sost and (**C**) Dkk1 mRNA levels. All values are expressed relative to reference group. Mean and individual mice values are shown. ^aaa^ *p* < 0.005 vs. Reference, ^b^ *p* < 0.05 and ^bbb^ *p* < 0.005 vs. CKD+NP. R.U., relative units vs. Reference. GAPDH was used as housekeeping gene.

The kidney Wnt/β-catenin pathway signaling showed a significant activation in the CKD+HP group (Figure 3A), which coincided with significantly higher mRNA levels of sclerostin (Sost) and Dickkopf-1 (Dkk1) compared to CKD+NP group (Figure 3B,C). At protein level, no significant changes were observed in Sost (CKD+NP: 0.96 ± 0.74 vs. CKD+HP: 1.52 ± 1.21 R.U.; *p* = 0.327), but Dkk1 levels were significantly higher in the CKD+HP group compared to the CKD+NP group (1.57 ± 1.06 vs. 0.55 ± 0.51 R.U., respectively; *p* = 0.048).

#### 3.1.3. Osteogenic Differentiation and Ca Deposition

**Figure 4:**
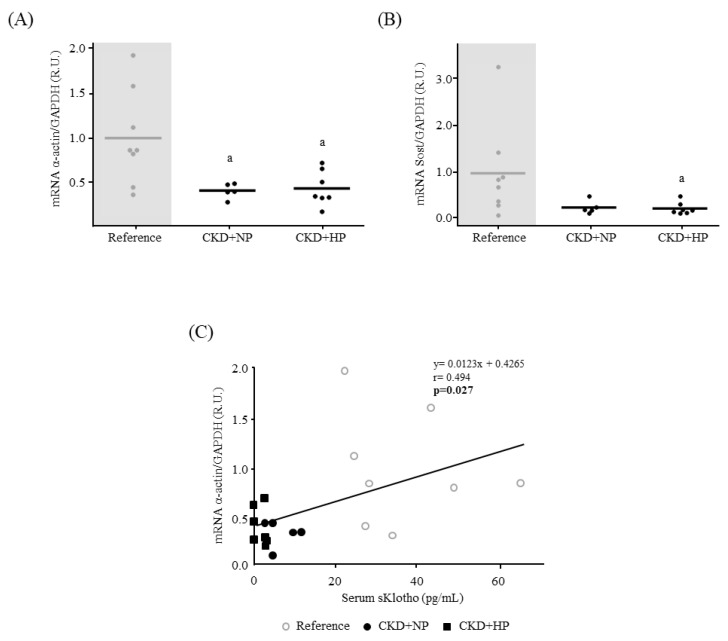
In vivo effects of chronic kidney disease (CKD) and dietary phosphorus intake on aortic osteogenic differentiation and components of the Wnt/β-catenin pathway. (**A**) α-actin and (**B**) Sost mRNA levels in abdominal aortas from Sham-operated (Reference) and CKD mice fed a normal (NP) or high phosphorus (HP) diet for 14 weeks. All values are expressed relative to reference group. Mean and individual mice values are shown. ^a^ *p* < 0.05 vs. Reference. R.U., relative units vs. reference group. GAPDH was used as housekeeping gene. (**C**) Correlation between α-actin mRNA levels and serum sKlotho in all groups. Each shape represents Reference (○), CRF+NP (●) and CRF+HP (■) values.

A significant lower vascular α-actin (Figure 4A) and Sost mRNA levels (Figure 4B) were observed in the CKD groups compared to the reference. A significant positive correlation between serum sKlotho and α-actin gene expression was also observed among the three experimental groups (r = 0.494, *p* = 0.027, Figure 4C).

Aortic Ca deposition was not observed in any group. The kidney showed Ca deposits in the two CKD groups having a significant negative correlation with kidney α-Klotho protein (r = −0.734; *p* = 0.004).

### 3.2. Patients CKD Stages 2 to 5 and Controls

The main clinical and biochemical parameters of patients are summarized in Table 2. No differences were found in the age and BMI between controls and the four CKD stages from CKD-2/3a stages to CKD-5. Serum creatinine showed a progressive increase, meanwhile the eGFR and urinary creatinine showed a significant decrease (Table 2). Proteinuria was significantly higher in CKD stages 3 to 5 (Table 2).

**Figure 5:**
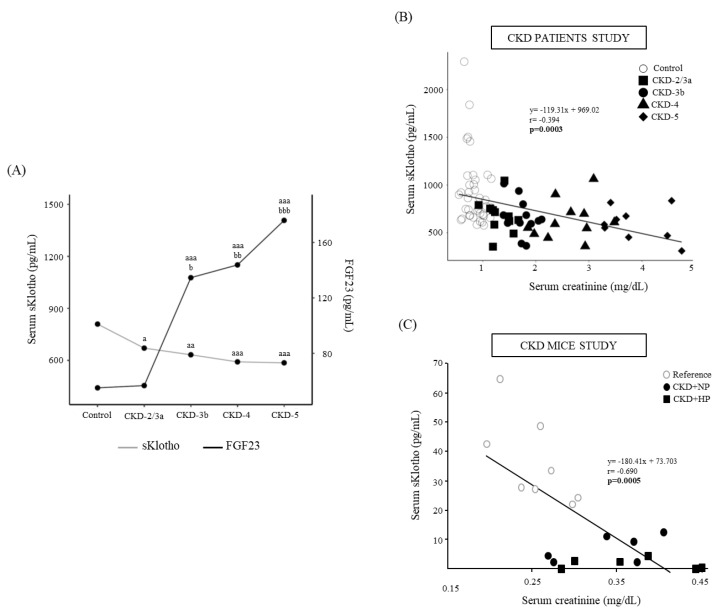
(**A**) Serum sKlotho and FGF23 in control subjects and chronic kidney disease (CKD) patients distributed according to eGFR from KDIGO guidelines. Median are shown. ^a^ *p* < 0.05, ^aa^ *p* < 0.01, ^aaa^ *p* < 0.005 vs. Control, ^b^ *p* < 0.05, ^bb^ p < 0.01, ^bbb^ *p* < 0.005 vs. CKD-2/3a. Correlation between serum sKlotho and serum creatinine in (**B**) the individuals described in (**A**). Each shape represents Control (○), CKD-2/3a (■), CKD-3b (●), CKD-4 (▲) and CKD-5 (♦) values; and in (**C**) Sham-operated mice fed normal phosphorus (NP) (Reference) and CKD mice fed NP or high phosphorus (HP) diet for 14 weeks, each shape represents Reference (○), CRF+NP (●) and CRF+HP (■) values.

No changes were observed in serum Ca and serum P showed a significant increase in CKD-5 (Table 2). Serum PTH and FGF23 showed a progressive increase from CKD-3b to CKD-5 (Table 2 and Figure 5A), and serum sKlotho showed a progressive decrease from CKD-2/3a to CKD-5 (Table 2 and Figure 5A). A significant negative correlation between serum sKlotho and serum creatinine was observed, in the same way as the results observed in the mice model (Figure 5B,C). A significant negative correlation between serum FGF23 levels and the estimated glomerular filtration rate (eGFR) was also found (r = −0.384, *p* = 0.011).

The urinary sKlotho significantly increased in CKD-2/3a stage (Table 2) and it remained at similar levels in CKD stages 3b to 5.

### 3.3. In Vitro Effect of sKlotho on VSMCs Signaling towards Osteogenic Differentiation

After 24 h of exposure of the primary VSMCs to CM and non-CM, no changes in Ca content, Osterix and Sost gene expression were observed and α-actin gene expression was significantly lower in the VSMCs cultured in CM (0.81 ± 0.11 vs. 1 ± 0.09, respectively; *p* = 0.002). The addition of sKlotho (50 ng/mL) did not modify the α-actin levels in the CM group (CM = 0.81 ± 0.11 vs. CM + sKlotho = 0.80 ± 0.11; *p* = 0.89).

**Figure 6:**
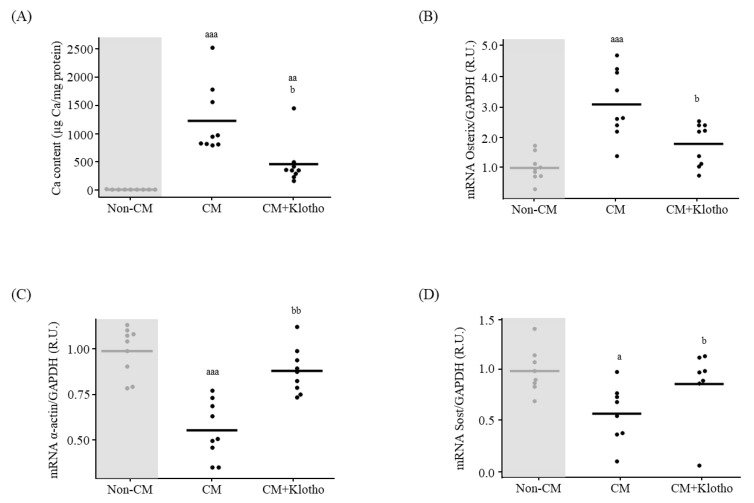
sKlotho effect on primary vascular smooth muscle cells (VSMCs) differentiation to osteoblast-like cells. (**A**) Ca content quantification, (**B**) Osterix (**C**) α-actin and (**D**) Sost mRNA levels in primary VSMCs exposed to non-calcifying medium (Non-CM), calcifying medium (CM) and CM+sKlotho for 3 days. All values are expressed relative to Non-CM condition as mean of 3 independent experiments by triplicates (horizontal lines). ^a^ *p* < 0.05, ^aa^ *p* < 0.01 and ^aaa^ *p* < 0.005 vs. Non-CM, ^b^ *p* < 0.05 and ^bb^ *p* <0.01 vs. CM. R.U., relative units vs. Non-CM. GAPDH was used as housekeeping gene.

After 72 h of exposure, Ca content and Osterix gene expression were higher and α-actin lower in the VSMCs exposed to CM (Figure 6A–C). When sKlotho (50 ng/mL) was added to the CM, the Ca content and Osterix gene expression were lower and α-actin higher compared to cells exposed to CM without sKlotho (Figure 6A–C). Sost mRNA levels were lower in cells exposed to CM compared to non-CM, and it increased significantly when sKlotho was added to the CM (Figure 6D).

### 3.4. Autophagy

#### 3.4.1. In Vivo: Autophagy Study in Aortas

**Figure 7:**
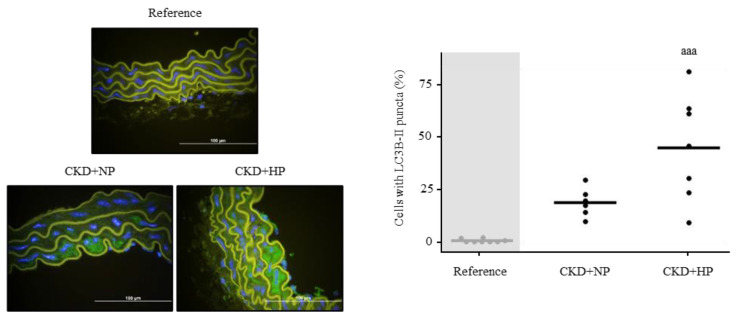
In vivo effects of chronic kidney disease (CKD) and dietary phosphorus intake on aortic autophagy. Representative images of LC3B-II and its quantification in aortas from Sham (Reference) and CKD mice fed NP or HP diet for 14 weeks. Each inset indicates the relative scale in µm. ^aaa^ *p* < 0.005 vs. Reference.

The CKD+NP and CKD+HP groups showed the percentage of cells with LC3B puncta was significantly higher compared to the reference group, reaching the highest levels in the CKD+HP group (Reference = 0.53 ± 0.86%, CKD+NP = 20.03 ± 7.41% and CKD+HP = 48.08 ± 27.25%; *p* = 0.0003; Figure 7).

#### 3.4.2. In Vitro: Autophagic Effect of sKlotho in VSMCs

**Figure 8:**
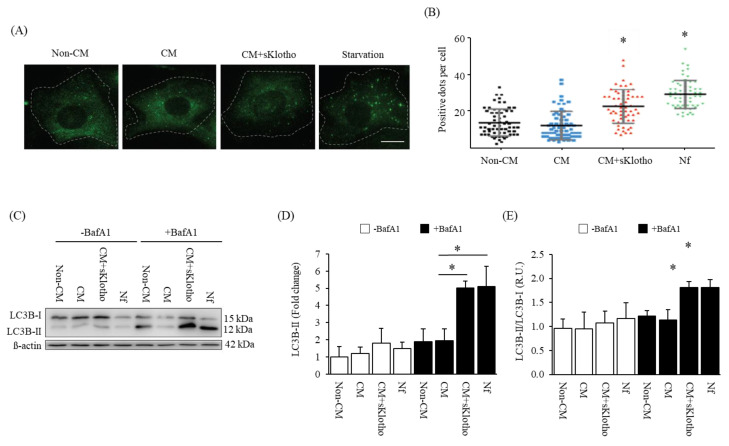
sKlotho effect on autophagy flux in primary vascular smooth muscle cells (VSMCs) (**A**) Representative images of LC3B-II from primary VSMCs exposed to Non-CM, CM and CM+sKlotho for 24 h. An autophagy positive control is shown (Starvation, Nutrient free medium, Nf). (**B**) LC3B-II puncta per cell quantification. All values are expressed relative to Non-CM condition. Mean and standard deviation values are shown. * *p* < 0.05 vs. Non-CM, positive LC3B-II puncta per cell. (**C**) Representative image of Western blot analysis and relative quantification of LC3B-II/LC3B-I ratio of primary VSMCs exposed to Non-CM, CM and CM+sKlotho for 24 h. An autophagy positive control is shown (Nf group). β-actin was used as a loading control. The Western blot image shows the experiment under –Bafilomycin and +Bafilomycin exposures. Relative quantification of LC3B-II protein (**D**) and LC3B-II/LC3B-I ratio (**E**). All values are expressed relative to Non-CM condition. Mean and standard deviation values are shown. * *p* < 0.05 vs. Non-CM, R.U: Relative Units.

After 24 h, primary VSMCs exposed to CM+sKlotho showed a greater number of LC3B puncta, in the same way as the positive control medium levels (Nutrient free, Nf). The cellular content of LC3B puncta was significantly greater compared to VSMCs exposed to CM without sKlotho (Figure 8A,B).

The measurement of the autophagic flux by the incubation with Bafilomycin A1 during the last 4 h of CM+sKlotho exposure, led to an increase in LC3B-II cellular levels comparable to that observed in the positive control medium (Nf) (Figure 8C–E). The increase in the autophagic flux obtained with CM+sKlotho was further confirmed in the rat VSMC cell line A7r5 (Figure S1).

## 4. Discussion

This study was designed to (a) confirm the potential role of sKlotho as an early biomarker of CKD-MBD, (b) to assess whether sKlotho is a reliable indicator of kidney α-Klotho content, (c) to deepen the effects of sKlotho on VSMCs osteogenic differentiation and (d) to evaluate if autophagy could be involved in the actions of sKlotho.

The in vivo experimental study showed that serum sKlotho was a reliable marker of kidney α-Klotho. In addition, it showed a significant decrease in sKlotho before the FGF23 increased, which was more pronounced in the CKD mice exposed to a high P diet (CKD+HP). In the aortas of the mice, serum sKlotho correlated with the loss of α-actin as a marker of the VSMCs contractile phenotype. The study in CKD patients confirmed that serum sKlotho decreased early during the course of CKD, and in the same way as in the mice study, this happened even before FGF23 levels started to rise. Finally, the in vitro study showed that sKlotho mitigated the VSMCs osteogenic differentiation and increased autophagy.

Klotho is synthesized mainly in the kidney, although it has also been detected in other tissues [34]. Cleavage of transmembrane Klotho results in the soluble form of the protein (sKlotho), which can be found in serum, urine and cerebrospinal fluid. It has been reported [9,35] that sKlotho levels decrease during the progression of CKD, though the value of sKlotho as an early and reliable biomarker of kidney α-Klotho production and CKD progression is still a matter of debate [36,37,38].

The mice model showed that after 14 weeks of a mild-moderate CKD (CKD+NP group), creatinine and PTH experienced a two-fold increase, which coincided with a significant reduction in serum but not urinary sKlotho. Importantly, the decrease in sKlotho occurred before the changes in other CKD-MBD biochemical parameters, such as Ca, P, calcitriol and even FGF23. Coincident with previous studies [27,39,40], the increase in P exposure (CKD+HP group), resulted in a further increase in serum P, PTH and FGF23, and a remarkable 18-fold significant decrease in sKlotho. Furthermore, serum sKlotho highly correlated with kidney α-Klotho content. Some authors have obtained the same correlation both in experimental models [41] and CKD patients [42], although not all agree [10]. This finding could have potential practical implications, because a blood sample could help to estimate the kidney α-Klotho content.

Urinary sKlotho levels decreased in both CKD mice groups following the same pattern of serum sKlotho, though the magnitude of the changes was lower than those observed in serum sKlotho. Importantly, urinary sKlotho decreased, but it did not correlate with kidney α-Klotho content, in contrast with the highly significant correlation observed between serum sKlotho and kidney α-Klotho (Figure 2D). These results indicate that serum sKlotho was a more precise marker of tissue Klotho content than urinary sKlotho. To our knowledge, there are no studies that have focused on urinary sKlotho as an indicator of kidney α-Klotho, just in serum sKlotho [10,41,42], probably because sKlotho removal through the kidney is not yet clear [13,43].

The reduction in serum and urinary sKlotho observed in the CKD groups coincided with high percentages of kidney fibrosis compared with controls, which goes in the same direction as other studies which showed that Klotho protected against renal fibrosis in mice [44,45]. In spite of the previous idea, there was no correlation between the severity of CKD and the degree of kidney fibrosis, indicating that, at least, in this CKD mice model, there was no relationship between the degree of fibrosis and the progression of the kidney disease. A significant eight-fold increase in urinary albumin was observed in the CKD+HP group, as a possible expression of the greater kidney damage induced by the CKD plus the high P exposure.

As kidneys are the main source of sKlotho, but at the same time the reduction in renal function can impair sKlotho removal in CKD, the interpretation of the values of urinary sKlotho is controversial [13,14]. sKlotho is a high-weight protein (130 kDa) that cannot be filtered out through the glomerular barrier. So far, two hypotheses have been proposed to explain the presence of sKlotho in the urine: Klotho tubular transcytosis and Klotho shedding by tubular proteases [13]. We could not evaluate if the sKlotho present in the urine of our mice proceeded from transcytosis or protease shedding, but we can confirm that the decline in sKlotho in serum and urine followed the same trend, though, serum sKlotho showed a remarkable higher decrease (18-fold), compared to urinary sKlotho (4-fold).

The study of patients with different CKD stages confirmed that serum sKlotho was the earliest CKD-MBD biomarker that showed changes and it was also observed in the experimental study, serum sKlotho decreased before FGF23 started to increase (Figure 5A). The clinical and experimental results of our study positioned serum sKlotho as the biomarker that changed earliest in CKD-MBD, which coincides with previous publications [9], despite the current controversy [11].

In CKD patients, urinary sKlotho followed an opposite pattern than serum sKlotho. It started to change in early stages of CKD, but it increased instead of decreased. A possible explanation for the discrepancy with the results in mice could be due to the different chronology, type and degree of renal insufficiency of mice and patients. The surgical experimental model of kidney damage may have proportionally affected more, or in a different way, the tubular structures compared with the spontaneous pathophysiological CKD alterations that occur in patients, in whom different glomerulo-tubular changes could be expected. The surgical-dependent morphological and structural kidney changes induced by the surgery in the mice model, may partly explain the difficulties in the tubular processing of sKlotho, and therefore impairing its removal. On the other hand, the increased excretion of sKlotho observed in the CKD patients may have favored the decrease in sKlotho observed in serum. Nevertheless, there are other studies that found no correlation between serum and urine sKlotho [46].

The results of the present study favor the practical value of serum compared to urinary sKlotho to predict α-Klotho kidney content, despite other studies having reported opposite results [9,10,47]. Another factor that can help to support the advantages of sKlotho is the fact that the latter is more unstable in urine than in serum, thus, the extraction technique, storage and sample processing are more determinant when measuring sKlotho in urine than in serum, and they can count as part of the discrepancies [14].

In addition, so far, there is no consensus about what it is the best technique to assess sKlotho, some authors claimed that sKlotho immunoprecipitation might be a better option than performing an ELISA assay, but the labor-intensive nature of immunoprecipitation requires further research, and it might not be extended to all laboratories [12]. In this study, ELISA assays were used to evaluate sKlotho, a technique which has shown successful performances in most studies [35,48,49].

It is important to stress that the strain of mice FVB/N used in our study is considered an appropriate mice model to study kidney damage [50,51], but not to study VC [52,53]. Therefore, as expected, none of the CKD mice developed VC, but Ca deposits in the kidneys were observed, showing a significant negative correlation with serum sKlotho and kidney α-Klotho protein, which agrees with previous publications [8].

Furthermore, despite the CKD mice not developing aortic calcification, important molecular changes related with the VSMCs changes that occur before VC is established were observed. In fact, the CKD groups showed lower aortic gene expression of Sost and α-actin, indicating a progressive loss of the VSMC contractile phenotype. In addition, α-actin positively correlated with serum sKlotho (Figure 4C), supporting the concept that sKlotho may be also a marker of changes in the VSMCs phenotype. This is of special relevance, as previous research has associated sKlotho levels with VC [54] and our results showed this association with the osteogenic differentiation, which takes place before VC.

sKlotho has been involved in the regulation of the Wnt/β-catenin signaling [30], and it has been considered one of the defensive natural mechanisms against VC [55]. Interestingly, the mice study displayed kidney signals of activation and inactivation of the Wnt/β-catenin pathway. The inactivation signals expressed by a high gene expression of Sost and Dkk1 may possibly reflect the negative feedback triggered by the kidney to prevent renal fibrosis and Ca deposits [19,40,56].

Autophagy has been proposed as a defensive mechanism against VC in CKD [21,22] and sKlotho is known to induce autophagy in several tissues [20,57]. Since our CKD mice displayed lower levels of sKlotho and to investigate the possible link of sKlotho and autophagy in the vasculature in CKD, the latter was evaluated in the aortas of the mice in which vascular-osteogenic differentiation changes had been initiated. Higher levels of autophagy were found in the CKD+HP group (Figure 7). In addition, the in vitro experiments showed that the addition of sKlotho to the CM significantly increased the autophagic flux, a finding that may indirectly indicate a protective role of sKlotho against VC (Figure 8).

Unfortunately, as autophagy is an extremely fast process [58], it was not possible to measure the autophagic flux after 72 h of culture, which was the time when the osteogenic differentiation was completed and the addition of sKlotho was able to prevent it.

The in vivo and in vitro effects of sKlotho found in our study are in keeping with other effects attributed to Klotho such as pro-autophagic actions in heart and kidneys [20], renoprotection [59], anti-apoptotic and anti-senescent actions in endothelial cells [60] and reduction in the negative effects of hyperphosphatemia on VC [8,61].

The present study has limitations. Despite the aggressive surgery carried out for obtaining renal damage in the rodent model, the level of CKD obtained might be assimilated to a CKD 3a-3b stages in humans. More specifically, CKD+NP mice could be said to mimic the human CKD 3a stage and the addition of a HP diet aims to increase the damage to mimic a CKD 3b stage. Thus, CKD+HP mice displayed a higher kidney damage and vascular impairment, discussed in previous paragraphs. However, it is necessary to take into account the inaccuracy to extrapolate the results obtained in an experimental model in mice to what is happening in the human CKD. Thus, the CKD mice and patients’ studies seem to be only partially extrapolated, as the spontaneous loss of renal function in CKD patients and the surgical reduction performed in the mice could have had different impacts on the mechanisms of sKlotho tubular removal [13,43]. Moreover, the reduced number of mice and the reduced amount of serum obtained in each mouse did not allow us to confirm the serum sKlotho results using other techniques such as immunoprecipitation. Despite the mentioned limitations, the study has an important strength, as the combination of experimental and patients’ results provided an interesting translational perspective.

## 5. Conclusions

In summary, the present clinical–experimental study showed that serum sKlotho declined early, before FGF23 increased, and it was a precise and reliable marker of the kidney α-Klotho content. In addition, the in vitro experiments showed that sKlotho recovered the vascular phenotype, reduced the osteogenic differentiation of the VSMCs and increased the autophagic signaling.

## Figures and Tables

**Table 1:** Weight and biomarkers of kidney dysfunction in the in vivo study.

	Reference (*n* = 8)	CKD+NP (*n* = 6)	CKD+HP (*n* = 7)
Final body weight (g)	32.21 ± 5.73	27.23 ± 1.89	**25.54 ± 2.86 ^aa^**
Creatinine (mg/dL)	0.25 ± 0.04	**0.34 ± 0.06 ^a^**	**0.37 ± 0.06 ^aaa^**
BUN (mg/dL)	23.19 ± 4.46	**41.67 ± 7.14 ^aaa^**	**36.45 ± 8.86 ^aaa^**
Calcium (mg/dL)	9.13 ± 0.56	9.56 ± 0.73	9.67 ± 0.46
Phosphorus (mg/dL)	5.29 ± 1.23	5.81 ± 0.79	**7.21 ± 1.66 ^a^**
PTH (pg/mL)	172.68 ± 58.35	**353.29 ± 121.95 ^a^**	**442.04 ± 219.37 ^aaa^**
FGF23 (pg/mL)	196.75 ± 132.13	186.00 ± 75.42	**1643.86 ± 892.96 ^aaa,bbb^**
1,25(OH)2D3 (pg/mL)	88.00 ± 29.25	104.71 ± 15.91	109.76 ± 20.30
sKlotho (pg/mL)	36.78 ± 14.81	**7.33 ± 4.56 ^aa^**	**1.85 ± 1.85 ^aaa^**
Calciuria (mg/24 h)	23.50 ± 15.41	26.50 ± 13.00	**9.90 ± 6.82 ^aaa,bb^**
Phosphaturia (mg/24 h)	7.20 ± 7.54	2.90 ± 6.40	**788.20 ± 483.95 ^aa,bbb^**
Urine albumin (µg/24 h)	32.14 [14.35–49.75]	12.96 [8.37–29.19]	**100.85 [37.62–118.32] ^b^**
Urine Klotho (pg/24 h)	2269 [1801–13,052]	1592 [1158–24,249]	**537 [373–857] ^a,b^**
Urine Klotho (pg Klotho/mg urine creatinine)	6384 [4988–64,903]	5876 [4100–13,483]	**2365 [377–2482] ^a,b^**

CKD, Chronic Kidney Disease; NP, normal phosphorus diet; HP, high phosphorus diet; Reference, Sham-operated fed NP. ^a^ *p* < 0.05, ^aa^ *p* < 0.01 and ^aaa^ *p* < 0.005, show differences between CKD either NP or HP and reference group; ^b^ *p* < 0.05, ^bb^ *p* < 0.01 and ^bbb^ *p* < 0.005, show differences between CKD+NP and CKD+HP. Values are expressed as Median [interquartile range] or mean ± standard deviation. Kruskal–Wallis and Dunn tests as post hoc analysis, with Bonferroni correction were used as statistical methods. Bold data represents significant differences.

**Table 2:** Clinical and biochemical parameters and sKlotho in the CKD patients.

	Control (*n* = 38)	CKD-2/3a (*n* = 11)	CKD-3b (*n* = 12)	CKD+NP (*n* = 6)	CKD+HP (*n* = 7)
Age (years)	66.50 ± 4.63	61.72 ± 10.11	67.33 ± 7.41	69.09 ± 8.94	68.78 ± 5.97
BMI (kg/m^2^)	27.19 ± 4.57	31.34 ± 4.70	26.79 ± 2.84	30.52 ± 6.60	26.74 ± 3.99
Creatinine (mg/dL)	0.83 (0.72–0.98)	**1.23 (1.19–1.50) ^aaa^**	**1.75 (1.63–1.84) ^aaa^**	**2.66 (2.30–2.95) ^aaa,b^**	**3.70 (3.41–4.48) ^aaa,bbb,c^**
eGFR (mL/min/1.73 m^2^)	81.5 (75–87.8)	**49.0 (47.0–53.0) ^aaa^**	**39.0 (33.8–40.3) ^aaa^**	**23.0 (20.5–24.5) ^aaa,b^**	**12.0 (12.0–13.0) ^aaa,bbb,c^**
Total protein (mg/dL)	70.70 ± 3.49	71.20 ± 3.08	69.45 ± 2.88	69.05 ± 3.56	68.50 ± 5.96
Calcium (mg/dL)	9.53 ± 0.27	9.53 ± 0.32	9.55 ± 0.37	9.66 ± 0.60	9.35 ± 0.45
Phosphorus (mg/dL)	3.64 (3.54–4.00)	**3.16 (3.54–3.53) ^a^**	**3.30 (2.69–3.47) ^aaa^**	3.41 (3.10–3.88)	**4.09 (3.84–4.87) ^a,bbb,ccc,ddd^**
PTH (pg/mL)	52.0 (41.5–60.5)	56.0 (43.0–75.0)	**86.5 (71.8–117.5) ^aaa^**	**114.0 (71.5–198.5) ^aaa,bb^**	**176.0 (155.0–212.0) ^aaa,bbb,c^**
1,25(OH)2D3 (pg/mL)	45.56 ± 11.55	38.80 ± 11.50	**29.13 ± 10.27 ^aaa^**	38.57 ± 17.89	**24.03 ± 12.14 ^aaa^**
FGF23 (pg/mL)	55.2 (46.3–64.7)	56.8 (52.1–62.8)	**134.6 (80.7–166.2) ^aaa,b^**	**143.8 (101.1–240.8) ^aaa,bb^**	**175.9 (141.2–1,689.0) ^aaa,bbb^**
sKlotho (pg/mL)	809.2 (680.3–1042.2)	**670.6 (604.2–746.0) ^a^**	**632.2 (599.8–713.0) ^aa^**	**591.4 (517.2- 706.8) ^aaa^**	**585.80 (469.40–674.20) ^aaa^**
Urine creatinine (mg/dL)	105.65 (74.63–150.80)	**44.80 (35.25–100.75) ^a^**	95.95 (57.58–114.38)	82.75 (56.75–95.73)	**47.20 (46.60–71.30) ^aaa^**
Proteinuria (g/L)	0.07 (0.05–0.09)	0.05 [0.03–0.16]	**0.44 (0.15–0.77) ^aaa,bb^**	**0.32 (0.09–0.94) ^aaa,bb^**	**0.58 (0.26–1.06) ^aaa,bbb^**
Urine Klotho (pg Klotho/mg urine creatinine)	433.4 (189.1–726.9)	**1143.1 (539.4–2220) ^aa^**	**1055.1 (910.8–2,103.5) ^aaa^**	**1003.3 (849.2–2819.2) ^aaa^**	**1099.7 (905.9–1503) ^aaa^**

CKD, Chronic Kidney Disease. ^a^ *p* < 0.05, ^aa^ *p* < 0.01 and ^aaa^ *p* < 0.001 vs. Control; ^b^ *p* < 0.05, ^bb^ *p* < 0.01, ^bbb^ *p* < 0.001 vs. CKD-2/3a; ^c^ *p* < 0.05, ^ccc^ *p* < 0.001 vs. CKD-3b; ^ddd^ *p* < 0.005 vs. CKD-4. Values are expressed as Median [interquartile range] or mean ± standard deviation. Kruskal–Wallis or ANOVA test and Dunn test or Tukey test as post hoc analysis, were used as statistical methods. Bold data represents significant differences.

## Data Availability

The data underlying this article will be shared upon reasonable request to the corresponding author.

## Supplementary Materials

The following supporting information can be downloaded at: https://www.mdpi.com/article/10.3390/nu15061470/s1: Table S1: Antibodies used for Western blot, immunohistochemistry, and immunofluorescence; Table S2: Pre-developed assays used for quantitative real time PCR (qPCR); Figure S1: sKlotho increases autophagy flux in A7r5 cells.
