# Supplementary material for: Serum and Urinary Soluble α-Klotho as Markers of Kidney and Vascular Impairment
Source: Nutrients. 2023 Mar 18;15(6):1470. doi: 10.3390/nu15061470 (PMC10057552; doi:10.3390/nu15061470)
Supplement: Supplementary file 1 [file nutrients-15-01470-s001.zip › nutrients-2227613-supplementary.pdf]

SUPPLEMENTARY MATERIAL

Table of contents:

1. <u>Table S1</u>: Antibodies used for western blot, immunohistochemistry and immunofluorescence.

2. <u>Table S2</u>: Pre-developed assays used for quantitative real time PCR (qPCR).

3. <u>Figure S1</u>. sKlotho increases autophagy flux in A7r5 cells.

Table S1: Primary antibodies and dilutions used in Western Blots (WB), immunohistochemistry (IHC) or immunofluorescence (IF).

Primary antibody	Procedure	Dilution	Commercial
Goat anti-Klotho	IHC, WB	1:200	R&D Systems (#AF1819)
Rabbit anti-non-phospho (Active) β-Catenin	IHC	1:100	Cell Signaling Technology (#8814)
Goat anti-Sost	WB	1:100	R&D Systems (#AF1589)
Rabbit anti-Dkk1	WB	1:500	Abcam plc (#ab109416)
Mouse anti-LC3B	IF	1:100	MBL Life Sciences (#M152-3)
Rabbit anti-LC3B	WB	1:1,000	Novus Biologicals (#NB600-1384)
Rabbit anti-GAPDH	WB	1:5,000	Santa Cruz Biotechnology (#sc-25778)
Mouse anti-ß-actin	WB	1:2,500	Sigma Aldrich (#A2228)

Table S2: Pre-developed assays used for quantitative real time PCR (qPCR).

Description	Gene symbol	Commercial
α-Klotho	Kl	Thermo Fisher Scientific (Mm00502002_m1)
Sclerostin	Sost	Thermo Fisher Scientific (Mm04208528_m1)
Dickkopf 1	Dkk1	Thermo Fisher Scientific (Mm00438422_m1)
Collagen type 1	Col1a1	Thermo Fisher Scientific (Mm00801666_g1)
α-actin	Acta2	Thermo Fisher Scientific (Mm00725412_s1)
Osterix	Sp7	Thermo Fisher Scientific (Mm04209856_m1)
A disintegrin and metallopeptidase domain 17	Adam17	Thermo Fisher Scientific (Mm00456428_m1)
Tumor necrosis factor alpha	Tnfα	Thermo Fisher Scientific (Mm01161290_g1)
Beclin 1	Becn1	Thermo Fisher Scientific (Rn00586976_m1)
Autophagy Related 5	Atg5	Thermo Fisher Scientific (Rn01767063_m1)
Microtubule-associated proteins 1A/1B light chain 3B	Map1lc3b	Thermo Fisher Scientific (Rn02132764_s1)
Glyceraldehyde-3-phosphate dehydrogenase	Gapdh	Thermo Fisher Scientific (Mm99999915_g1)

<u>Figure S1</u>: sKlotho increases autophagy flux in A7r5 cells. (A) Representative images of LC3B-II immunofluorescence from A7r5 cells exposed to Non-CM, CM and CM+sKlotho for 24 hours. An autophagy positive control is shown (Starvation, Nutrient free medium, Nf). (B) LC3B-II puncta per cell quantification of the previous immunofluorescence from A7r5 cells exposed to Non-CM, CM, CM+sKlotho and nutrient free medium (Nf, positive control) for 24 hours. All values are expressed relative to Non-CM condition. Mean and standard deviation values are shown. *$p<0.05$ vs. CM, positive LC3B-II puncta per cell. (C) Representative image of Western blot analysis and relative quantification of LC3B-II/LC3B-I ratio of primary VSMCs exposed to Non-CM, CM and CM+sKlotho for 24 hours. An autophagy positive control is shown (Nf group). β-actin was used as a loading control. The Western blot image shows the experiment under –Bafilomycine and +Bafilomycine exposures. Relative quantification of LC3B-II protein (D) and LC3B-II/LC3B-I ratio (E) of A7r5 cells exposed to Non-CM, CM and CM+sKlotho for 24 hours. ß-actin was used as a loading control. All values are expressed relative to CM condition. Mean and standard deviation values are shown. $^{*}p<0.05$ *vs.* CM, R.U: Relative Units.

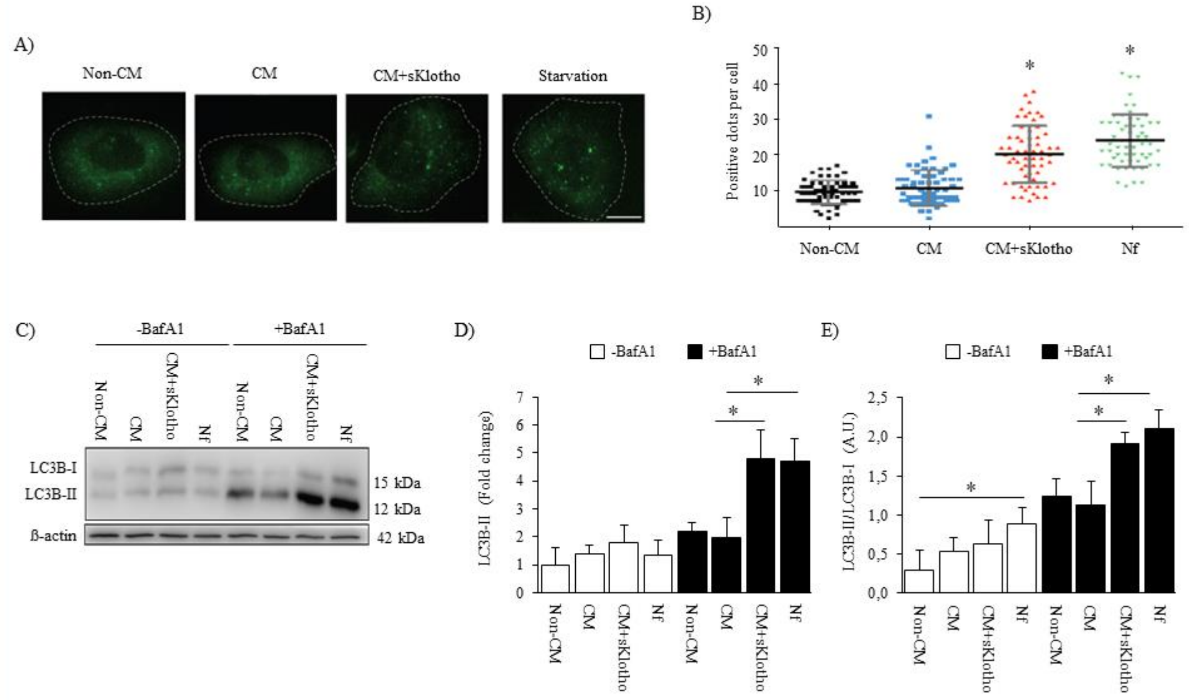

Figure S1 (autophagy in A7r5 cells)
